# Ethanol precipitation with cooling enables rapid purification of gelatin methacryloyl (GelMA) with increased yield and preserved solution-state organisation

**DOI:** 10.1038/s41598-026-51741-2

**Published:** 2026-05-08

**Authors:** Paritat Thaitalay, Ashish Pandit, Lauma Ievina, Julija Bulatova, Antons Sizovs, Janis Locs

**Affiliations:** 1 Institute of Biomaterials and Bioengineering, Faculty of Natural Sciences and Technology, Riga Technical University, Paula Valdena Street 3-K1, Riga, Latvia; 2 Baltic Biomaterials Centre of Excellence, Headquarters at Riga Technical University, Riga, Latvia; 3 Latvian Institute of Organic Synthesis, 21 Aizkraukles St, Riga, Latvia

**Keywords:** Biotechnology, Chemistry

## Abstract

Gelatin methacryloyl (GelMA) is a widely used photocrosslinkable biomaterial, yet routine laboratory synthesis is constrained by multi-day dialysis required to remove cytotoxic methacrylic anhydride (MAA) and methacrylic acid (MA). Here we introduce a simple post-reaction precipitation of GelMA in ethanol (EtOH) as a faster purification route. EtOH precipitation combined with cooling increased the isolated yield after purification (up to + 17.5% versus the control) and markedly accelerated impurity removal, reaching commercially relevant residue benchmark (< 30 ppm) within 2 days, whereas the conventional route required 7 days under identical dialysis conditions. Importantly, CD and SEC indicated that prolonged dialysis altered GelMA solution-state organization while EtOH precipitation followed by only 2 days of dialysis better produced material more similar to the parent gelatin. Hydrogels prepared from control and EtOH-treated GelMA displayed comparable swelling, rheology, compressive response, and indirect cytocompatibility with MC3T3-E1 cells. This accessible EtOH-assisted workflow reduces purification time without sacrificing functionalization or hydrogel performance and may improve batch-to-batch consistency for laboratory-scale GelMA production.

## Introduction

Protein-based biomaterials have attracted significant attention in biomedical research due to their tunable chemistry, favourable mechanical properties, and inherent bioactivity^1^. Gelatin, a denatured form of collagen, is a biocompatible and biodegradable polymer commonly used in a variety of biomedical applications, including bone tissue regeneration^2^. However, native gelatin forms thermally reversible gels with limited mechanical strength, restricting its use under physiological conditions^3^. To address this limitation, methacrylic anhydride (MAA) is widely used to introduce methacryloyl groups into gelatin, forming gelatin methacryloyl (GelMA). The initial synthesis protocol was reported by Van Den Bulcke et al. in 2000^4^, using phosphate-buffered saline (PBS) as the reaction medium and MAA as the functionalizing agent. Since then, numerous efforts have been made to improve the efficiency, reproducibility, and application-specific performance of GelMA.

GelMA has been extensively synthesised in both neutral phosphate-buffered saline (PBS, pH 7.4) and alkaline carbonate-bicarbonate (CB, pH 9) systems. PBS has long been used as the conventional buffer, whereas CB buffer has recently gained attention due to its ability to achieve a higher degree of functionalization (DoF) in GelMA compared with PBS^5^. They demonstrated that sequentially adjusting the pH above the isoelectric point (IEP, ~ 7–8) of type A gelatin could enhance the DoF of GelMA (from 47 to 87% in PBS, and 78% to 100% in CB buffer). A similar outcome was also reported by Hu et al.^6^, who confirmed that stepwise pH modulation effectively increases the DoF in both PBS and CB systems. However, this protocol requires labour-intensive manual pH adjustments, and the DoF achieved is highly dependent on operator skill. Such complexity could limit its scalability for industrial production. Shirahama et al. later showed that GelMA could be synthesized in CB buffer without sequential pH adjustments^7^, but despite this advantage, PBS remains more favourable for cell-based applications. In fact, PBS-buffered GelMA has been reported to support higher survival rates of bone marrow-derived mesenchymal stem cells^8^ and human lung adenocarcinoma cell lines^9^ in vitro compared with CB buffer. Furthermore, PBS is widely used in laboratory practice due to its physiological pH, osmotic balance, and established safety for living cells. Thus, although CB buffer offers a route to increase DoF, the use of PBS provides a more biologically relevant and practically reliable system, particularly when cytocompatibility and translational feasibility are prioritized.

In addition to buffer system, purification is a crucial step to ensure the cytocompatibility of synthesized GelMA. Dialysis remains the standard method for removing unreacted MAA and byproduct methacrylic acid (MA), both of which are cytotoxic. Classic dialysis uses a semipermeable membrane to remove these small-molecule impurities and typically requires 1–2 weeks to complete^10^. This prolonged process is labour-intensive and time-consuming. Several alternative approaches have been developed to shorten the purification period, including single-phase synthesis (3 days)^11^, microwave (MW) irradiation (1 day)^10^, and tangential flow filtration (up to 10 h)^12^. However, these methods often require specialized equipment or technical expertise, making them less accessible in low-resource laboratories and limiting their reproducibility across different settings. For classic dialysis, a wide range of conditions has been reported in the literature^13–18^, with durations from 1.5 to 7 days, temperatures between 40 and 50 °C, and water changes occurring every 4 h to once daily. Purification periods of 5–7 days appear to be commonly accepted and effective in many studies^19–24^. Despite the widespread use of dialysis in GelMA purification, no standardised protocol exists to define the optimal conditions, particularly the duration required to effectively remove residues. Although a commercial benchmark of < 30 ppm has been reported for X-Pure^®^ GelMA (Rousselot Inc.)^25^, this threshold has not been widely evaluated or adopted in academic settings.

Therefore, this study investigates whether a modified GelMA synthesis route can reduce purification time while achieving residual MA/MAA levels comparable to this commercial standard. Rather than altering reaction chemistry or employing specialized purification systems, we introduce simple ethanol pretreatment applied after conventional methacrylation to accelerate removal of residual reactants. The degree of functionalisation (DoF) of GelMA was assessed by proton nuclear magnetic resonance (^1^H-NMR) for a control sample and by ninhydrin colourimetric assay across all experimental groups. To confirm the chemical structure, Fourier-transform infrared spectroscopy (FTIR) analysis was performed to compare bonding features between samples. Additionally, UV spectroscopy was used to monitor the efficiency of the purification process and to evaluate whether the EtOH pretreatment and shortened dialysis protocol effectively removes residual impurities within a reduced timeframe. To test whether the modified synthesis route affects cytocompatibility, indirect cell metabolic activity was assessed using the CellTiter-Blue^®^ assay.

## Materials and methods

### Materials

Gelatin from porcine skin (type A, 300 g bloom, CAS. 9000-70-8), methacrylic anhydride (MAA, CAS. 760-93-0), phosphate buffered saline (PBS, Ref. N. P4417-100TAB), deuterium oxide (D_2_O, CAS. 7789-20-0), ninhydrin (CAS. 485-47-2), and 2-hydroxy-4′-(2-hydroxyethoxy)-2-methylpropiophenone (I2959, CAS. 106797-53-9) were purchased from Sigma Aldrich, Germany. Ethanol (EtOH, CAS. 64-17-5). The dialysis membrane with molecular cut-off 12–14 kDa was sourced from Spectrum lab™, USA. To avoid interference from impurities, Milli-Q water was used in all experimental procedures.

### Synthesis of gelatin methacryloyl

**Fig. 1:**
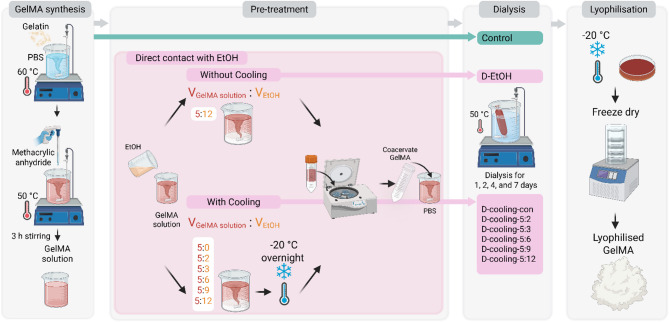
Schematic representation of the synthesis procedures used to prepare GelMA under different experimental conditions (created using BioRender.com).

Gelatin (10 g) was dissolved in 100 mL PBS at 60 °C under stirring (400–500 rpm) until homogeneous (20–60 min). After cooling to 50 °C, 1.4 mL MAA was added dropwise, and the reaction was maintained at 50 °C for 3 h, during which methacrylation was completed. The resulting GelMA solution was diluted with 400 mL of preheated (50 °C) PBS. EtOH was therefore introduced only after methacrylation had occurred, rather than during the functionalisation reaction itself. The solution was then subjected to different EtOH pretreatment protocols: control (no treatment), and direct contact (with and without cooling), as illustrated in Fig. 1. Each condition was then dialysed against Milli-Q water at 50 °C for 1, 2, 4, or 7 days, with daily water changes (10 mL water/1 mL GelMA solution). The dialysed GelMA solution was frozen at − 20 °C overnight, freeze-dried for 72 h, and stored at − 20 °C until further use. Lyophilization duration was identical for all samples and was not varied as an experimental parameter. Samples from each pretreatment group were labelled as presented in Table 1.

**Table 1:** GelMA composition and various pretreatment conditions.

Sample series	V_GelMA solution_: V_EtOH_ ratio	Pretreatment conditions
Control	5:0	–
D-EtOH	5:12	Without cooling
D-cooling-con	5:0	Cooling
D-cooling-5:2	5:2	Cooling
D-cooling-5:3	5:3	Cooling
D-cooling-5:6	5:6	Cooling
D-cooling-5:9	5:9	Cooling
D-cooling-5:12	5:12	Cooling

### Chemical characterization of gelatin methacryloyl

#### Proton nuclear magnetic resonance (^1^H-NMR)

Gelatin and control GelMA sample (7 mg/mL) were prepared by dissolving in D_2_O at 37 °C until homogeneous (30–60 min). ^1^H-NMR spectra were acquired at 37 °C on a DD2 500 MHz spectrometer (Agilent Technologies, Santa Clara, CA, USA) with 5 s relaxation delay. The degree of methacryloyl functionalisation was calculated using the following Eq. (1):

1 $$\:\mathrm{D}\mathrm{o}\mathrm{F}\mathrm{\%}\:=\:\left(1\:-\frac{\int\:\:lysine\:methylene\:proton\:of\:GelMA\:\left(3\:ppm\right)}{\int\:\:lysine\:methylene\:proton\:of\:gelatin\:\left(3ppm\right)}\right)\:\times\:\:100\mathrm{\%}$$

#### Yield calculation

GelMA yield (%) was calculated as the ratio of the mass of freeze-dried GelMA obtained after dialysis to the estimated mass of gelatin present in the reaction mixture prior to dialysis, according to the following Eq. (2):

2 $$\:\mathrm{Y}\mathrm{i}\mathrm{e}\mathrm{l}\mathrm{d}\:\left(\mathrm{\%}\right)\:=\:\frac{{W}_{\mathrm{lyophilised\:}\mathrm{GelMA}}}{{W}_{\mathrm{initial\:}\mathrm{gelatin}}}\:\times\:\:100\mathrm{\%}$$

where $\:\:{W}_{\mathrm{lyophilised\:GelMA}}\:\:$is the weight of lyophilized GelMA and $\:\:{W}_{\mathrm{initial\:}\mathrm{gelatin}}\:\:$ is the weight of starting gelatin used during GelMA synthesis.

#### Ninhydrin assay

The DoF of GelMA was also quantified using a colourimetric ninhydrin assay, modified from the protocol of Zatorski et al.^26^. A gelatin stock solution (10 mg/mL in PBS) was serially diluted to 0–7.5 mg/mL to establish a standard curve. GelMA samples were prepared at 7 mg/mL in PBS. A ninhydrin stock solution (8.8 mg/mL) was prepared in 70% EtOH and mixed with gelatin or GelMA solutions at a volume ratio of 1:8. The mixtures were heated at 100 °C for 25 min, cooled to room temperature, and transferred to a 96-well plate. Absorbance was measured at 570 nm using a Tecan Infinite M Nano microplate reader (Tecan, Männedorf, Switzerland), and the DoF was calculated using Eq. (3)

3 $$\:\mathrm{D}\mathrm{o}\mathrm{F}\mathrm{\%}\:=\left(1-\:\frac{Apparent\:sample\:concentration}{No\mathrm{min}al\:sample\:concentration}\right)\:\times\:\:100\mathrm{\%}$$

#### Fourier-transform infrared spectroscopy (FTIR)

The FTIR analysis was performed using a Nicolet iS50 FTIR spectrometer (Thermo Fisher Scientific, USA) equipped with an ATR mode. Spectra were collected in transmittance mode over 4000–400 cm^−1^ at 4 cm^−1^ resolution with 64 scans. A background spectrum was recorded before each measurement and subtracted from the sample spectrum.

#### UV–Vis spectroscopic analysis

UV–Vis’s spectrophotometer (Thermo Scientific, Evolution 300, Madison, WI USA) was used to quantify the concentration of MA residual remaining in the GelMA solution by analyzing the dialysed water samples collected from outside the dialysis membrane at different dialysis time points. A standard calibration curve for MA was prepared by serially diluting MA in Milli-Q water to concentrations ranging from 0 to 100 µg/mL. UV spectra for all standards and samples were recorded over a wavelength range of 190–400 nm, with a scan speed of 240 nm/min and a 1 nm interval. Quartz cuvette with 10 mm path length was used. Absorbance at 205 nm was used for calibration, and linearity between absorbance and MA concentration was confirmed via linear regression analysis. The resulting regression equations were then applied to calculate MA content in each dialysed water sample.

#### Size-exclusion chromatography (SEC)

Material samples were dissolved in eluent pre-warmed to 37 °C and agitated for 2 h to ensure complete solubilization. The resulting solutions were filtered through 0.2 μm polyethersulfone (PES) membrane spin filters. Size-exclusion chromatography (SEC) was performed on an OMNISEC system (Malvern Panalytical, UK) equipped with RESOLVE and REVEAL modules (RI, RALS, LALS, UV–Vis detectors). Separations were carried out on a PL aquagel-OH 30 column (8 μm, 300 × 7.5 mm; Agilent Technologies) at 37 °C and 22 °C, using 0.2 M NaNO_3_ buffered to pH 7.0 with 0.01 M Na_2_HPO_4_/NaH_2_PO_4_ as the mobile phase at a flow rate of 1.0 mL/min. Data were processed using OMNISEC software (v11.40). Refractive index and molecular weight distribution traces were exported from OMNISEC, normalized, and plotted in GraphPad Prism 10 (v10.3.1; GraphPad Software, LLC).

#### Circular dichroism (CD)

Circular dichroism (CD) spectra (180–300 nm) were collected on a Jasco J-1500 CD spectropolarimeter equipped with a Peltier temperature-controlled cell holder and an immersible temperature probe. Measurements were performed in a 0.1 cm pathlength quartz cuvette at 4, 22, and 37 °C. Samples were prepared by dissolving materials in Milli‑Q water pre-warmed to 37 °C and agitating for 2 h to ensure complete solubilization. Solutions were then filtered through 0.2 μm PES membrane spin filters and equilibrated at the measurement temperature (4, 22, or 37 °C) for at least 2 h prior to data acquisition to allow conformational equilibration of gelatin chains. After CD data acquisition, to account for potential material loss during filtration, gelatin concentrations were measured using refractometry. CD spectra were normalized by the measured concentrations. CD spectra were plotted using GraphPad Prism 10 (v10.3.1; GraphPad Software, LLC).

### Characterisation of gelatin methacryloyl hydrogel

#### Rheological performance

Strain- and temperature-dependent rheology were performed using a Thermo HR-20 Hybrid rheometer (TA Instruments, USA). Lyophilized GelMA was dissolved in PBS (10% w/v) at 37 °C until fully homogeneous. 110 µL GelMA solution was placed on a 25 mm parallel plate geometry with a 0.15 mm gap. Strain sweeps were conducted from 1–10,000% strain at 22 °C with a constant angular frequency of 1 Hz to observe the linear viscoelastic (LVE) region. Temperature sweeps were performed from 40 to 4 °C (cooling down) and 4 to 40 °C (heating up) at 6.5 °C/min, with a constant 1% strain and 1 Hz angular frequency. The sol-gel transition temperature (T_sol-gel_) was defined as the crossover point of the storage (G′) and loss (G″) moduli.

#### Swelling behavior

I2959 photoinitiator was dissolved in PBS (0.1% w/v) at 70 °C for 1 h. Lyophilized GelMA was then dissolved in this solution (10% w/v) at 37 °C until homogeneous. 400 µL GelMA solution was cast into cylindrical molds (10 mm diameter, 5 mm height) and photocrosslinked for 4 min under UV light (365 nm, 20 W; Alonefire SV44, Shenzhen ShiWang Technology Co., Ltd., China). Crosslinked GelMA hydrogels were incubated in PBS (20 mL/sample) at 37 °C with gentle agitation (100 rpm). PBS was changed daily. The swelling % of hydrogels was determined at defined time points and calculated using Eq. (4):

4 $$\:\mathrm{S}\mathrm{w}\mathrm{e}\mathrm{l}\mathrm{l}\mathrm{i}\mathrm{n}\mathrm{g}\:\left(\mathrm{\%}\right)\:=\:\frac{{{W}_{\mathrm{t}}-W}_{\mathrm{0}}}{{W}_{\mathrm{0}}}\:\times\:\:100\mathrm{\%}$$

where$\:\:{W}_{\mathrm{t}}\:\:$ is the weight of hydrogel at time t and$\:\:{W}_{\mathrm{0}}\:\:$ is is the initial weight prior to immersion in PBS.

#### Compression test

The compressive mechanical properties of crosslinked GelMA hydrogels were evaluated using a universal testing machine equipped with a 500 N load cell (Zwick/Roell Z020, Zwick GmbH & Co. KG, Germany). Hydrogel specimens were prepared following the same protocol described for the swelling experiment. Compression tests were performed at a crosshead speed of 1 mm/min until 70% strain was reached.

### In vitro cellular testing

#### Material extract preparation

2 mL of each material were sterile filtered using a 3 mL syringe (Plastipak, BD, New Jersey, USA) and 0.2 μm syringe filters (Whatman, GE Healthcare, United Kingdom). 100 µL of material groups were crosslinked in 2 mL microcentrifugation tubes for 4 min with UV light (365 nm, 20 W; Alonefire SV44, Shenzhen ShiWang Technology Co.,Ltd., China). This sample was incubated in 1 mL of α-MEM (Sigma-Aldrich, Saint Louis, MO, USA) supplemented with 10% fetal bovine serum (FBS; Sigma-Aldrich, Saint Louis, MO, USA) and 1% penicillin/streptomycin (P/S; Sigma-Aldrich, Saint Louis, MO, USA) for 24 h in 37 °C. After the incubation, extracts were collected and frozen in − 20 °C until cell culture experiments.

#### Cell expansion

MC3T3-E1 cells were thawed, seeded in T-75 flasks, and cultured in α-MEM (Gibco™, Thermo Fisher, USA) supplemented with 10% FBS and 1% P/S. Cultures were incubated at 37 °C and 5% CO_2_, with medium changed every 2 days until 80–90% confluence was reached.

#### Metabolic activity assay

MC3T3-E1 cells were counted using a Fluidlab R-300 (Anvajo, Germany) and seeded in a 96-well plate at a density of 1 × 10^4^ cells per well. After overnight attachment in α-MEM supplemented with 10% FBS and 1% P/S, the medium was replaced with 100 µL material extracts described previously. For positive control, full media with 10% DMSO was used; for negative control, cells were grown in full media. Cell viability was assessed after 24 h. CellTiter-Blue^®^ (CTB) Cell Viability Assay (Promega, Madison, WI, USA), and fluorescence was measured with a Tecan Infinite M Nano microplate reader (Tecan, Männedorf, Switzerland).

#### Live/dead staining

After CTB assay the wells were washed with PBS and incubated for 30 min in 37 °C with PBS with Ethidium homodimer (MedChemExpress, New Jersey, USA) (4 µM working concentration) and Calcein-AM (Sigma-Aldrich, Saint Louis, MO, USA) (2 µM working concentration) according to manufacturers’ protocol. Imaging was done using confocal laser Scanning microscopy (CLSM, LSM 900, Zeiss) in 5× maginfication. Ex/em wavelengths of Calcein-AM is 494/517 nm and for Ethidium homodimer 528/617 nm.

### Replicates and statistical analysis

GelMA yield values were obtained from a single synthesis batch per condition (*n* = 1) and are reported as individual measurements. DoF and UV–Vis measurements were performed on samples from one synthesis batch per condition with triplicate technical measurements and are reported as mean ± SD to reflect assay variability. Swelling (*n* = 3), rheological (*n* = 3), and compression (*n* = 7) tests were conducted using independently prepared hydrogel specimens. For in vitro analysis all groups were tested in triplicates (*n* = 3). Statistical significance for cell metabolic activity was tested using Kruskal–wallis test. Statistical analysis was conducted using GraphPad Prism 10.2.3. (GraphPad Software Inc., USA).

## Results and discussion

**Fig. 2:**
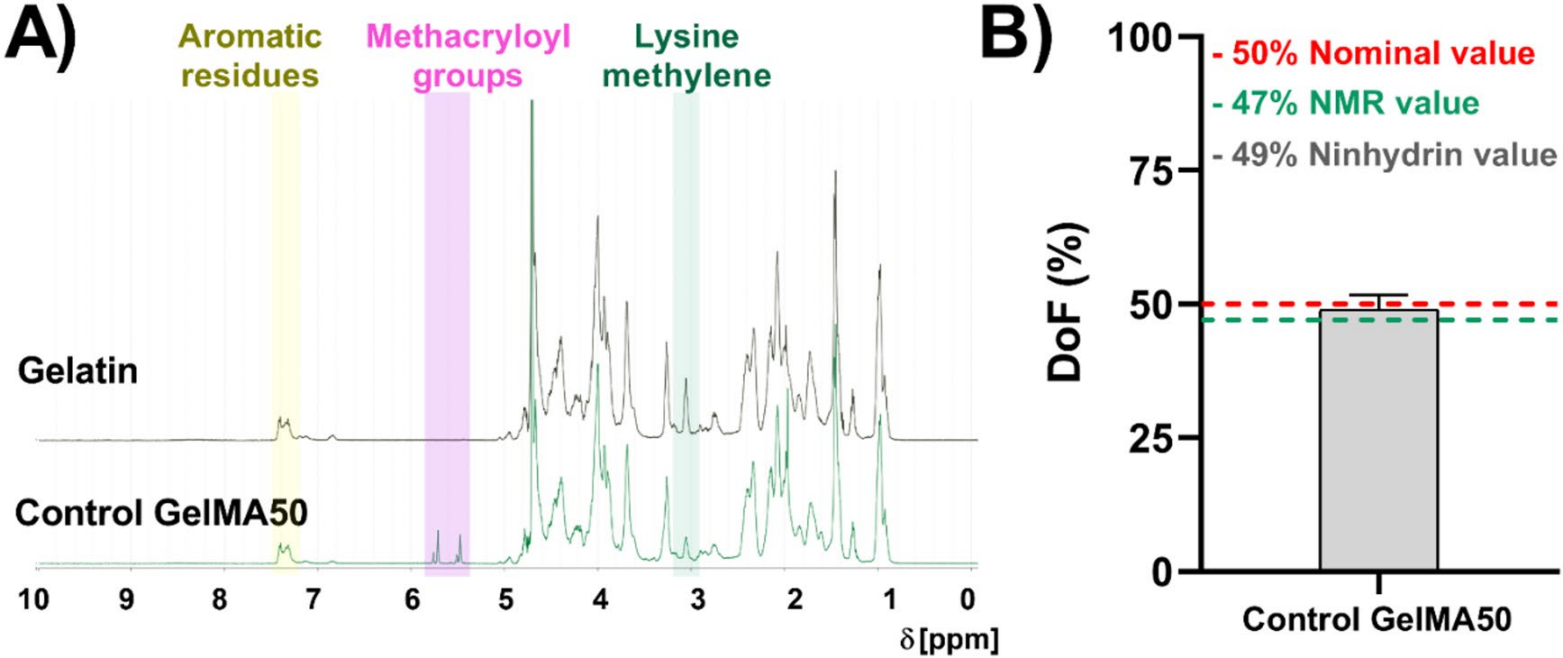
^1^H-NMR spectra of pure gelatin and control GelMA (**A**), degree of functionalisation (DoF%) of control GelMA analyzed by ^1^H-NMR and ninhydrin assay (**B**).

^1^H-NMR spectra of the pure gelatin and control GelMA sample were analysed. As shown in Fig. 2A, methacrylation was indicated by a decrease in lysine methylene peaks (3.0–3.1 ppm) and the appearance of methacryloyl group peaks (5.4–5.8 ppm) in the control GelMA, consistent with previous reports^27^. Peak areas corresponding to lysine methylene protons in both pure gelatin and GelMA were normalised to the constant phenylalanine signal (7.2–7.5 ppm), as commonly described in the literature^28^. Using Eq. (1), DoF% was determined to be 47.3%, which was in close agreement with the nominal DoF (50%) and the result from the ninhydrin colourimetric assay (48.9 ± 2.8%, Fig. 2B). Based on this consistency, the ninhydrin assay was chosen as the primary method to evaluate DoF% in other sample conditions.

**Fig. 3:**
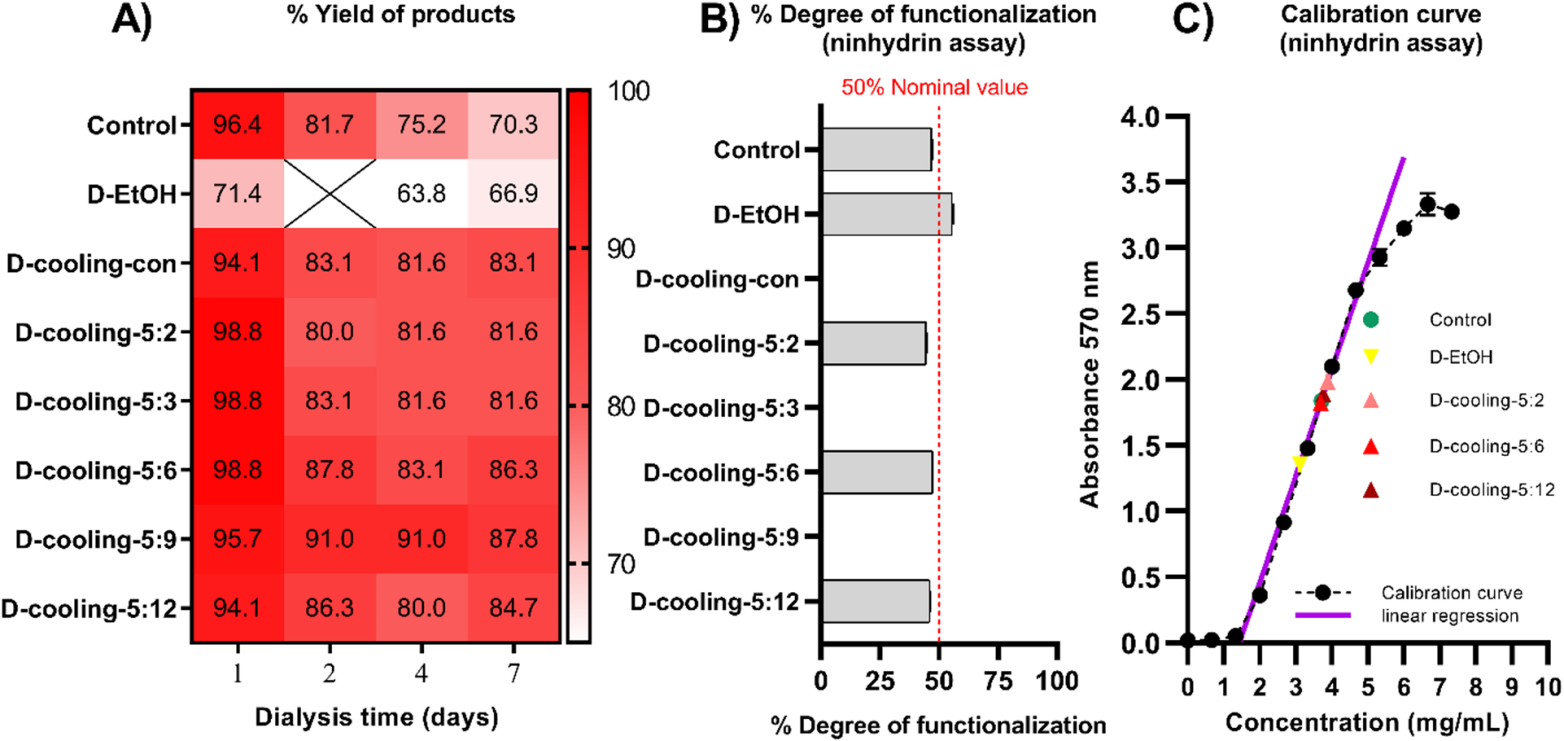
Heatmap showing GelMA product yield from various synthesis methods throughout the dialysis process from 1 to 7 days (single batch per condition; *n* = 1) (**A**), degree of functionalisation (DoF%) of selected GelMA conditions at 7 days dialysis, determined using the colourimetric ninhydrin assay (mean ± SD from technical triplicates of a single batch) (**B**). Calibration curve from serially diluted gelatin standards (0–7.5 mg/mL) used for DoF% quantification (**C**).

**Fig. 4:**
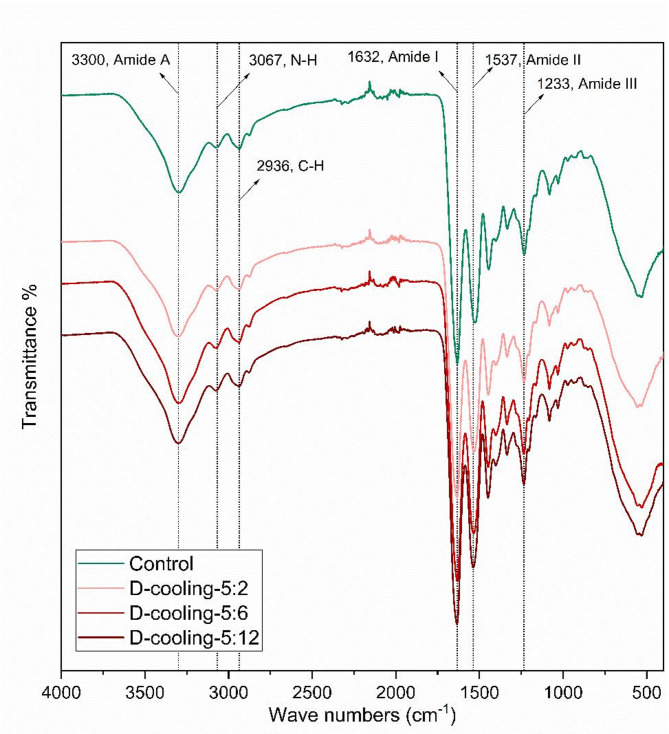
FTIR spectra of selected GelMA conditions at 7 days of dialysis.

Yield is increased by EtOH precipitation with cooling. After 7 days of dialysis, the control synthesis yielded 70% GelMA (Fig. 3A), while direct EtOH contact without cooling gave 67%. In contrast, applying an overnight cooling step (with or without EtOH) increased the isolated yield to ~ 82–88% across the tested EtOH: GelMA-solution ratios. This likely reflects enhanced physical gelation and chain association at low temperature, which can reduce diffusion of soluble fragments through the dialysis membrane and thereby decrease material loss during purification^29,30^. While GelMA yield values were obtained from a single synthesis batch per condition, consistent trends across multiple EtOH pretreatment ratios (5:2, 5:3, 5:6, 5:9, 5:12) and purification time points (1, 2, 4, 7 days) indicate the method’s reliability. Even at early time point, EtOH-treated samples consistently maintained higher yields (> 80%) compared with the control (day 4: 75%; day 7: 70%). These observations suggest that, despite being based on a single batch, the EtOH pretreatment approach reproducibly enhances material recovery. Future work should include multi-batch replicates to fully confirm robustness. EtOH pretreatment does not compromise methacrylation. For representative cooling-assisted conditions (EtOH: GelMA solution ratios of 5:2, 5:6, and 5:12), the ninhydrin assay indicated DoF values close to the nominal 50% (Fig. 3B,C). FTIR spectra of all selected samples showed the expected amide bands (Amide I–III) and no qualitative differences attributable to EtOH pretreatment (Fig. 4), supporting that EtOH introduced after methacrylation affects yield and purification efficiency rather than the extent of functionalization. All spectra exhibited characteristic peaks at 1233, 1537, and 1632 cm^−1^, corresponding to C–N stretching/N–H bending (Amide III), N–H bending (Amide II), and C = O/C–N stretching (Amide I)^31^, respectively. Additional peaks were also observed at 2936, 3067, and 3300 cm^−1^, which are attributed to saturated C–H stretching, N–H stretching, and O–H/N–H stretching (Amide A)^32^, respectively.

EtOH-induced coacervation/precipitation of gelatin has been widely reported at sufficiently high EtOH contents^33^, and has been exploited to form gelatin nanoparticles^34^, nanosphere aggregates, and networks^35^. Here we leveraged this phase-separation behaviour after methacrylation to remove low-molecular-weight reactants and by-products (MAA/MA) into an EtOH-rich supernatant before dialysis, with the goal of shortening the overall purification time^19–24^, and maintaining hydrogel functional properties. Previous microwave-assisted GelMA synthesis study^10^ observed phase separation in the obtained GelMA solution and attributed the precipitates to residual methacrylic acid based on DSC analysis, however, no quantitative evaluation of residuals was performed, and the resulting hydrogel properties were altered compared with conventional GelMA. In contrast, our EtOH precipitation is a designed purification step that efficiently removes low-molecular-weight MAA/MA byproducts (< 30 ppm) while preserving polymer chain integrity and degree of functionalization. This approach maintains hydrogel performance and demonstrates a clear mechanistic and practical advantage over previously reported incidental precipitation phenomena. To quantify purification kinetics, we monitored methacrylate-containing residues released into the dialysate over 7 days using UV–Vis spectroscopy. Jongprasitkul et al.^36^ previously employed UV–Vis spectroscopy to demonstrate GelMA purification by monitoring MAA residues in dialysed water. Comparing with a 10 w/v% MA reference (absorption maxima 200–250 nm), they observed disappearance of MAA peaks after 5 days of dialysis with 2–3 water changes per day. However, to the best of our knowledge, no quantitative investigation has reported MA/MAA residue concentrations relative to the acceptable level (< 30 ppm^25^ described for a commercially available product. While UV–Vis has previously been used to qualitatively follow GelMA dialysis, our approach applies a calibration curve to enable direct comparison between synthesis conditions. Because the assay quantifies residues in the dialysate rather than in the final lyophilized GelMA, it primarily reports relative purification efficiency and direct residual analysis of the final material remains an important next step.

**Fig. 5:**
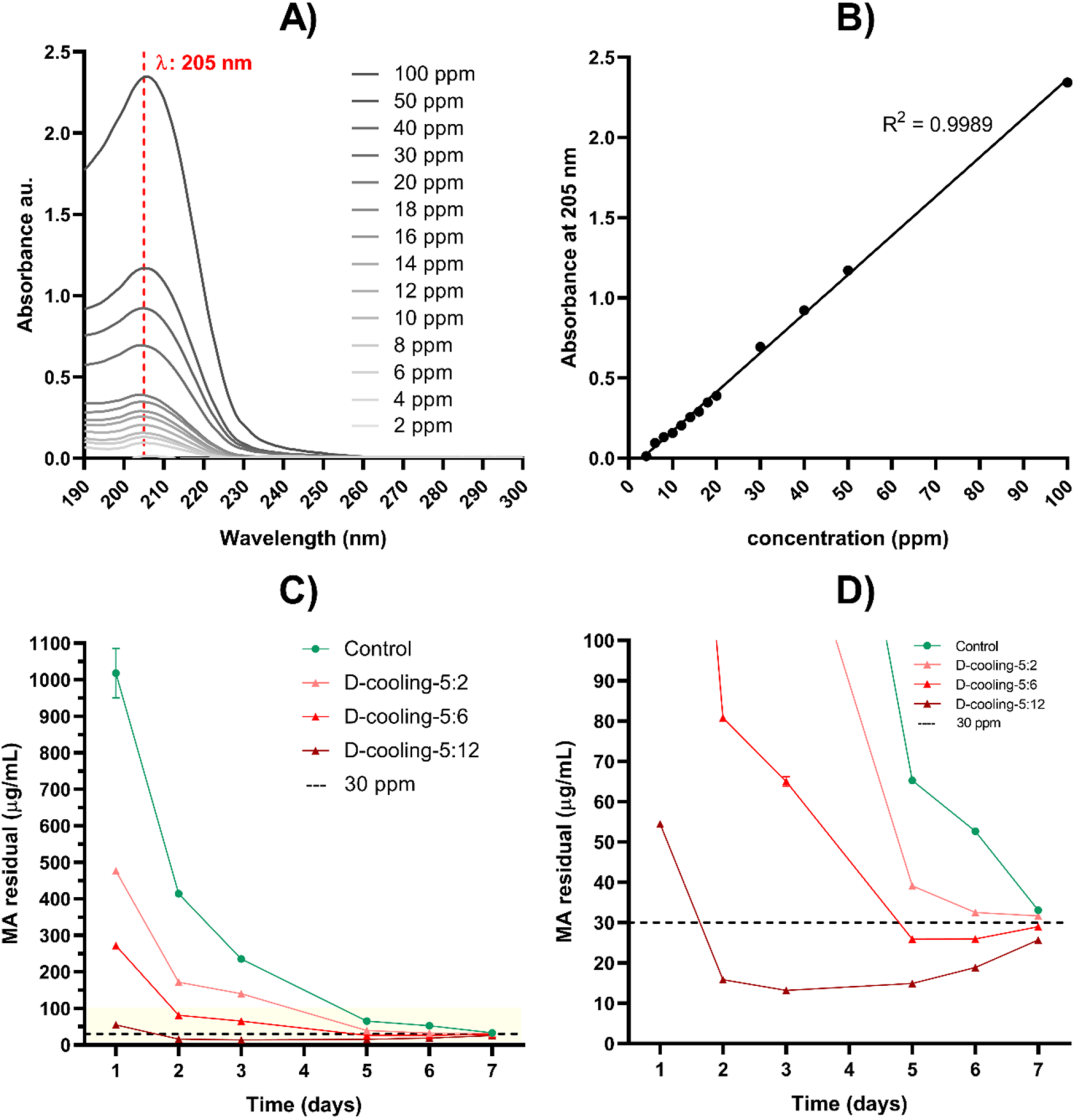
UV–Vis spectroscopic analysis of MA/MAA residues during GelMA purification. UV spectra of MAA standard solutions (0–100 µg/mL) showing a concentration-dependent increase in absorbance at 205 nm (**A**). Calibration curve of MAA with excellent linearity (**B**). Residual MA/MAA concentrations in dialysed water samples from different synthesis conditions over 7 days of dialysis (daily water change) (**C**). Magnified view of panel C (0–100 ppm range) highlighting differences in low-level MA/MAA residues among samples (**D**).

UV spectra of MA standards (0–100 µg/mL) exhibited a concentration-dependent increase in absorbance at 205 nm (Fig. 5A), enabling construction of a calibration curve with excellent linearity (R^2^ ≥ 0.99; Fig. 5B). Dialysate samples from all conditions were quantified against this curve. Notably, samples synthesised via direct EtOH contact with cooling reached the < 30 ppm benchmark more rapidly than the control GelMA. In particular, the D-cooling-5:12 condition fell below 30 ppm after just 2 days, whereas the control required the full 7 days to achieve the similar level (Fig. 5C,D). This accelerated purification may be explained by two factors. First, as described earlier, EtOH-induced coacervation facilitated removal of MAA residues into the EtOH phase. Second, when the mixture was cooled to − 20 °C, GelMA precipitated from solution due to its temperature- and EtOH/H_2_O mixture- dependent solubility, whereas EtOH remained in the liquid state (melting point ≈ − 114 °C). Following centrifugation, the precipitated GelMA was collected, and the EtOH-rich supernatant containing residual impurities was discarded.

**Fig. 6:**
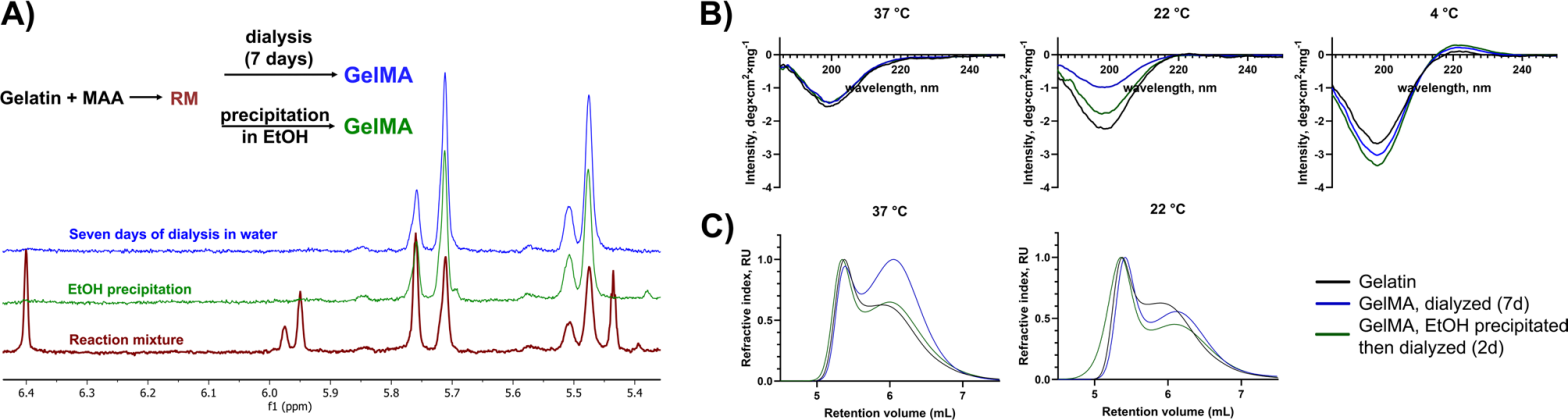
Physicochemical characterization of GelMA. Vinyl region of ^1^H NMR spectra of the unpurified reaction mixture (RM) (red), GelMA obtained by EtOH precipitation prior to dialysis (D-cooling-5:12, green), and GelMA obtained by conventional 7-day dialysis (blue) (**A**). Circular dichroism spectra at 4, 22, and 37 °C of starting gelatin (black), GelMA dialysed for 7 days (blue), and GelMA precipitated in EtOH and subsequently dialysed for 2 days (green) (**B**). Refractive index (RI) traces from SEC for the same materials (**C**).

¹H-NMR spectra (Fig. 6A) of the reaction mixture after the GelMA synthesis is completed, expectedly contained vinyl peaks attributable to MAA/MA. These impurities are effectively removed by the classical 7 days of dialysis against water, but importantly—no MAA/MA vinyl peaks were observed for GelMA obtained after precipitation in ethanol (D-cooling-5:12 prior to dialysis). Although the sensitivity of ^1^H-NMR might not reveal ppm levels of MAA/MA, this result still clearly demonstrates high efficiency of ethanolic precipitation for removal of most of the MAA/MA.

DoF and FTIR confirm successful functionalization, but they do not capture potential changes in molecular weight distribution or solution-state organisation introduced by different purification workflows. Therefore, we compared starting gelatin with two representative products (i) conventionally purified GelMA (7-day dialysis) and (ii) EtOH-precipitated GelMA purified by only 2 days of dialysis using circular dichroism (CD) and size-exclusion chromatography (SEC) (Fig. 6B,C).

Circular dichroism (Fig. 6B) revealed temperature-dependent differences in solution-state organization. At 37 °C, all materials showed broadly similar spectra dominated by a negative peak at 197–199 nm, consistent with a predominantly disordered (random-coil) state in warm solution. Upon cooling to 22 and 4 °C, gelatin exhibited the expected increase in ellipticity and a peak at 222 nm associated with triple helix of collagen-like order that forms during physical gelation. Importantly, the EtOH-precipitated GelMA purified by only 2 days of dialysis tracked closely with gelatin across temperatures, whereas the 7-day dialysed GelMA showed altered temperature response, suggesting that prolonged dialysis at elevated temperature can unforeseeably alter self-assembly properties of GelMA chains.

Size-exclusion chromatography (Fig. 6C) supported CD observations at the level of molecular size distribution and macromolecular association. SEC measurements acquired at 22 and 37 °C, indicate that EtOH‑precipitated GelMA after shortened 2 day dialysis exhibits size distribution broadly comparable to the starting gelatin, with two populations corresponding to aggregated fraction (earlier elution, with peak at ~ 5.4 mL) and non-aggregated/monomeric fraction (later elution, with peak at ~ 6.1 mL). No additional early-eluting signal was observed that would indicate new type of aggregation behavior after EtOH treatment, and no new low-molecular weight population was observed that would be consistent with extensive chain scission into substantially smaller fragments. In contrast, the 7-day dialysed GelMA displayed a visibly altered RI signal shape at 37 °C with increased relative signal corresponding to lower molecular weight population (gelatin monomer). Together with CD results, this suggests that EtOH precipitation allows rapid impurity removal while better preserving gelatin-like solution-state organization, whereas extended dialysis may introduce subtle changes that are not apparent from DoF and FTIR alone. This provides a structural rationale for favouring the EtOH + cooling workflow not only from reducing purification time perspective, but also to avoid altering the innate self-assembly properties of parent gelatin.

**Fig. 7:**
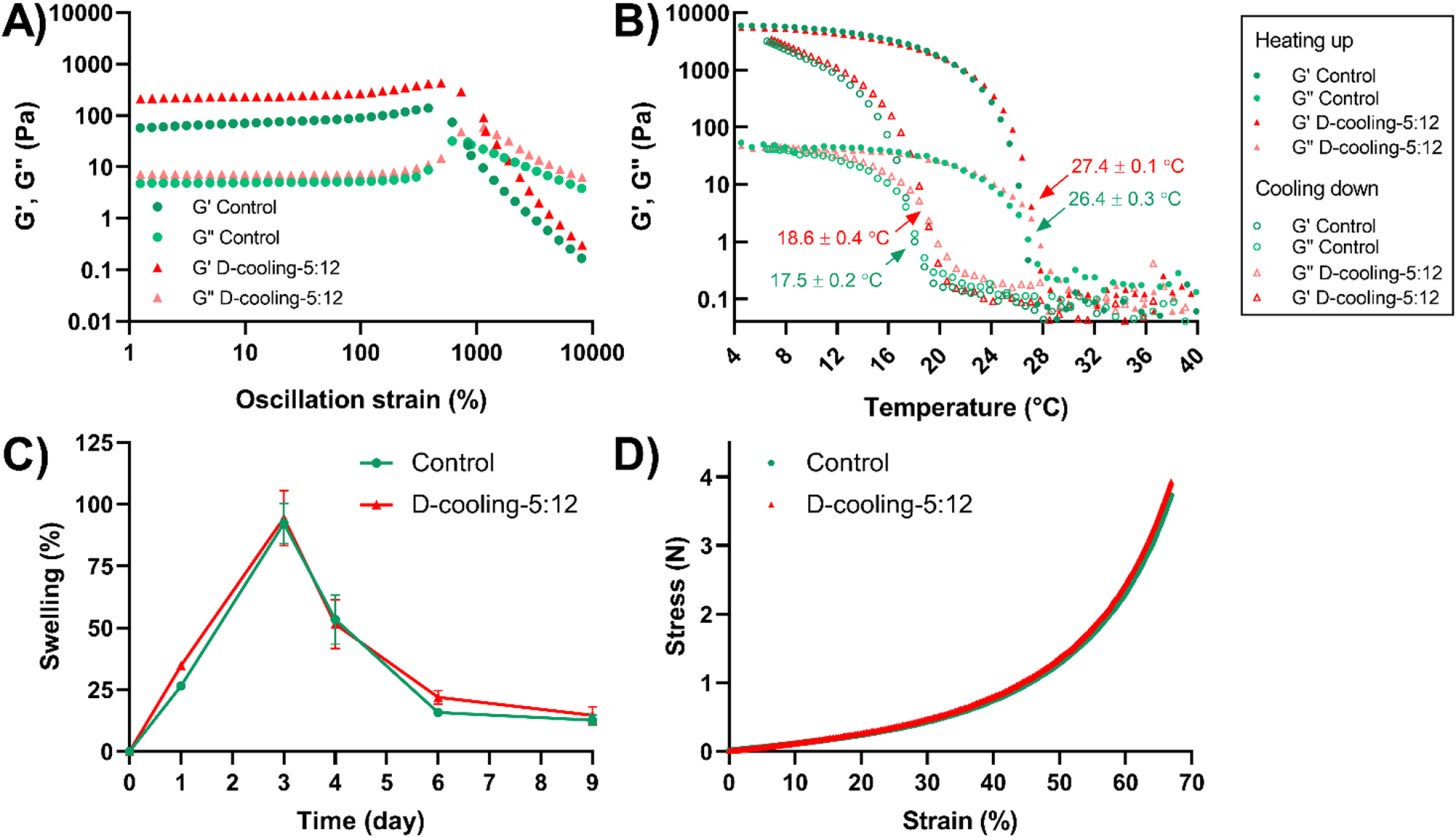
Functional characterisation of control and EtOH-pretreated GelMA (D-cooling-5:12) hydrogels. Strain sweep showing the linear viscoelastic region (**A**), temperature sweep indicating gel–sol transition temperature (**B**), swelling behavior over time in PBS (**C**), and compressive stress–strain curves (**D**). Data are presented as mean ± SD (*n* = 3 for rheology and swelling, *n* = 7 for compression).

Hydrogel functional performance is maintained. To test whether the shortened purification workflow translated into differences at the hydrogel level, we prepared GelMA hydrogels from control GelMA and EtOH-pretreated GelMA (D-cooling-5:12) under identical formulation and photocrosslinking conditions. Strain sweeps and temperature sweeps indicated comparable linear viscoelastic behaviour and sol-gel transition temperatures, respectively (Fig. 7A,B). Likewise, swelling kinetics in PBS and compressive stress-strain responses showed no meaningful differences between groups under the conditions tested (Fig. 7C,D). The similarity in hydrogel properties can be attributed to the preservation of the degree of functionalisation, as confirmed by the ninhydrin assay, which governs crosslinking density. In addition, SEC and CD analyses indicated no significant changes in molecular weight distribution or chain conformation after EtOH pretreatment. These results demonstrate that structural preservation at the molecular level translates directly to consistent rheology, swelling, and mechanical behavior in the hydrogels, validating the functional relevance of the purification strategy. Together, this suggests that EtOH-assisted purification removes low-molecular-weight species without altering the polymer network, resulting in comparable hydrogel performance.

**Fig. 8:**
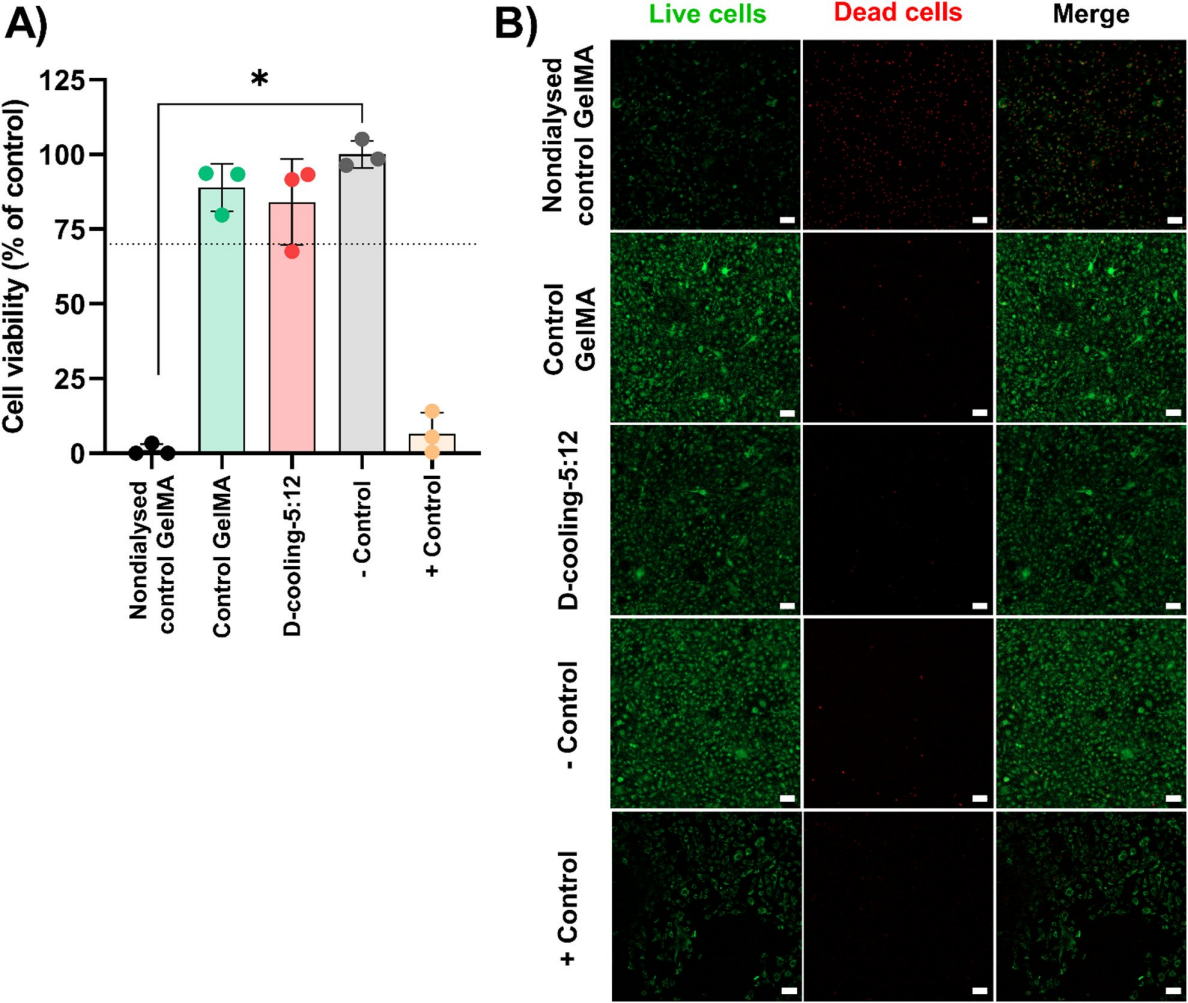
Indirect cytocompatibility assessment of GelMA hydrogels according to ISO 10,993 extract method. Cell metabolic activity measured by CellTiter-Blue Cell Assay after exposure to extraction media (0.1 g mL^−1^) from nondialysed control GelMA, dialysed control GelMA, and EtOH-pretreated GelMA (D-cooling-5:12), compared with negative and positive controls (**A**). Live/Dead fluorescence images of cells cultured with extracts from the same conditions (**B**). Scale bar = 100 μm. Data are presented as mean ± SD (*n* = 3). Statistical significance was indicated as follows: *p* < 0.05 (*).

To test whether the new method affects cytocompatibility, indirect cell metabolic activity tests with CellTiter-Blue^®^ assay were done after cells were incubated in material extracts in full culture media for 24 h. As shown in Fig. 8A, D-cooling-5:12 shows sustained cytocompatibility (84.1%) with MC3T3-E1 cell line after 24 h of incubation and, according to ISO-10,993^37^, can be classified as biocompatible. Similar results were observed in live/dead staining with Calcein-AM and Ethidium homodimer (Fig. 8B). Both control GelMA and D-cooling-5:12 showed high intensity of live, green-fluorescent cells and low number of dead cells. Fluorescent imaging shows uniformly arranged cells, with control GelMA cells having slightly more spread out morphology and higher green intensity than the rest of the groups. Some cells in D-cooling-5:12 group also show spread out morphology, but the intensity of the green fluorescence is slightly lower than the control groups. Minor variations in fluorescence intensity observed between conditions fit the results acquired from metabolic activity measurements with CTB assay. Data from our cytocompatibility experiment match other results from the literature, meaning that this modified GelMA synthesis protocol does not decrease materials cytocompatibility^8,38–40^. While this indirect approach confirms the absence of acute cytotoxic effects associated with residuals, further studies involving direct cell encapsulation within GelMA hydrogels will be required to fully evaluate cell-material interactions in 3D environments.

Unlike previously reported GelMA optimisation strategies that rely on modified reaction chemistries, specialised reactors, or continuous filtration systems, the present work introduces a simple post-synthesis ethanol pretreatment step to accelerate purification without altering the conventional synthesis conditions or compromising hydrogel performance. As summarised in Table 2, existing approaches, including single-phase system^11^, microwave-assisted synthesis^10^, flow-based reactors^12,40,41^, pH-controlled systems^9^, and tangential flow filtration, primarily achieve reduced processing time through changes in reaction environments or the use of specialized equipment, often accompanied by altered hydrogel properties or limited residual verification. Although ethanol-induced gelatin precipitation and coacervation have been previously reported, these strategies have largely focused on bulk gelatin recovery or phase separation, rather than the selective removal and verification of methacrylation byproducts in GelMA systems. In contrast, the ethanol pretreatment presented here is applied after GelMA synthesis, enabling efficient removal of residual reactants while preserving the polymer network formed during conventional methacrylation. Importantly, this study combines quantitative indirect residual assessment (UV–Vis, < 30 ppm, within acceptable limits) with qualitative direct confirmation by ¹H NMR, an aspect that is inconsistently addressed or absent in many previously reported protocols (Table 2). Moreover, the proposed method achieves an increased yield while maintaining swelling behavior, rheological response, and mechanical properties comparable to conventionally purified GelMA, alongside demonstrated cytocompatibility. Notably, this combination of accessible implementation, validated purification, and preserved hydrogel performance is not simultaneously demonstrated in existing GelMA optimisation strategies. The integrated evidence from yield, DoF, SEC/CD structural analyses, to hydrogel mechanics and cytocompatibility provides a cohesive mechanistic understanding of why EtOH pretreatment with cooling is effective and safe. These features distinguish the present ethanol pretreatment approach from existing solvent-assisted or specialised reactor-based purification strategies and highlight its practicality for routine GelMA preparation. Together, these effects highlight EtOH pretreatment with cooling as a promising approach to minimising dialysis time, offering practical advantages for laboratory-scale GelMA production, as evidenced by the day 2 D-cooling-5:12 sample showing residual MAA below the 30 ppm threshold. These findings suggest potential relevance for future scale-up, although industrial applicability was not directly assessed in this study.

**Table 2:** Comparison of reported GelMA synthesis and purification optimisation strategies.

Method	Yield	Purification time	Residual verification	Hydrogel performance vs. conventional	Biocompatibility	Key advantages	Key limitations
Single-phase system ^11^	N/A	3 days	Qualitative (¹H-NMR, direct)	Reduced sol–gel transition temperature	N/A	Reduced MA usage and synthesis time	Altered hydrogel properties
Microwave-assisted synthesis ^10^	N/A	1 day	Not reported	↑ Gel fraction, ↑ mechanical strength, ↓ swelling	N/A	Greatly reduced synthesis time	No residual confirmation
Tangential flow filtration (TFF)^40^	~ 90%	Several hours	Qualitative (¹H-NMR, direct: residual methacrylic acid detected)	Comparison with conventional GelMA not reported, but batch consistency emphasized	Huh7.5 cells (non-toxic)	Fast, reproducible purification	Requires complex equipment
Tangential flow filtration (TFF)^12^	N/A	10 h	Indirect (conductivity)	Comparison with conventional GelMA not reported	NIH-3T3 fibroblasts (non-toxic)	Reduced purification time	No direct residual confirmation; complex setup
Flow reactor synthesis ^41^	66% (+ 16%)	3 days	Not reported	Comparison with conventional GelMA not reported	Primary human endometrial stromal cells (non-toxic)	Increased yield and DoF	No residual confirmation
pH-controlled synthesis^9^	N/A	7 days	Not reported	↓ swelling, ↑ G′ (gelatin type B)	A549 cells (non-toxic)	Reduced MA usage	Altered hydrogel properties; no residual confirmation
This work: EtOH pretreatment	86% (+ 16%)	2 days	Quantitative (UV–vis, indirect, < 30 ppm) + qualitative (¹H-NMR, direct)	Comparable swelling, rheology, and mechanics	MC3T3-E1 cells (ISO-10993 compliant, non-toxic)	Reduced purification time, increased yield, preserved properties, simple implementation	Further in-depth biological evaluation suggested

## Conclusion

Here we show that ethanol precipitation combined with an overnight cooling step can be introduced as a simple post-reaction treatment to accelerate GelMA purification and improve isolated yield without compromising functionalization or hydrogel performance. Across the tested EtOH: GelMA-solution ratios, the degree of functionalization remained close to the nominal ~ 50% (ninhydrin assay, FTIR), while SEC and CD analyses indicated that polymer chain conformation and molecular weight distribution were preserved. Additional SEC and CD analyses further supported the structural similarity of EtOH-treated and control GelMA. Notably, only the direct contact–EtOH method combined with a cooling step enhanced the final product yield after 7 days of dialysis, resulting in a 11.3–17.5% higher yield relative to the control across the tested EtOH-to-GelMA solution ratios. UV–Vis monitoring of the dialysate (reported as methacrylic acid equivalents) demonstrated that the optimized condition (D-cooling-5:12) reached the commercially relevant benchmark of < 30 ppm within 2 days under once-daily water changes, whereas the control required 7 days under the same dialysis conditions. SEC and CD indicated that prolonged dialysis can alter GelMA solution-state organization, while ethanol precipitation followed by only 2 days of dialysis produced material more similar to the parent gelatin. Importantly, hydrogels prepared from control and EtOH-treated GelMA showed comparable rheology, swelling, compressive response, and indirect cytocompatibility with MC3T3-E1 cells, demonstrating that preserved structure, together with enhanced yield, translates directly to maintained hydrogel performance. Together, these findings illustrate how ethanol precipitation with cooling simultaneously preserves chemical structure, enhances purification efficiency, and maintains hydrogel properties, establishing a cohesive mechanistic and practical rationale for its use as a laboratory-scale GelMA purification strategy.

## Data Availability

The datasets used and/or analyzed in the present study are available from the corresponding author upon reasonable request.
